# On the Beneficial Effect of Creosote on Warty Excrescences

**Published:** 1856-01

**Authors:** Geo. Rainey

**Affiliations:** Lecturer on Anatomy and Demonstrator of Microscopical and Surgical Anatomy at St. Thomas’s Hospital


*On the Beneficial Effect of Creosote on Warty Excrescences.* By Geo. Rainey, Esq., M. R. C. S., Lecturer on Anatomy and Demonstrator of Microscopical and Surgical Anatomy at St. Thomas's Hospital.

It occurred to me, about three years ago, whilst making some experiments on the effects of creosote in preventing and arresting cell-growth, in solutions of substances remarkable for their rapid development of fungi, that it might possibly be applied with advantage in that obstinate form of porrigo called "porrigo lupinosa," especially if taken internally at the same time. This idea I mentioned some time afterwards to Dr. Bristowe, assistant-physician to St. Thomas's Hospital, who thought it sufficiently feasible to deserve a trial, and accordingly he resolved to employ it in the next case of this complaint which came under his care. He has not, however, since met with a case of this disease; I therefore determined to try it in some other disease resulting from excessive or abnormal cell-development, and last summer I took the opportunity of trying its effect on an obstinate warty excrescence on the finger, which was spreading very fast, in which case its action was very decisive, and to all appearance specific.

In order to secure the full effect of the creosote on the disease, after applying it freely to the part, I prevented its removal by a piece of adhesive plaster put several times round the finger, which was allowed to remain for two days. On removing the plaster, a visible change had taken place in the character of the surface of the excrescence, which now, in the place of being dry and hard, had become so soft and friable as to admit of being broken down by the slightest friction of the finger. The daily application of the creosote was, however, still continued until the remains of the wart had become of a horny consistence, after which, in about a fortnight, it desquamated, leaving the part beneath perfectly healthy.

The creosote, in this case, caused no pain or uneasiness, or any symptom which indicated an escharotic action on the affected part, but seemed to act entirely by destroying that excessive and abnormal cell-development which is the essential character of this form of disease.

In these excrescences there is what pathologists call hypertrophy of the epidermis. The epidermic cells also retain their nuclei and power of cell-growth longer than the normal epidermic cells of the stratum Malpighii of the surrounding parts; and the transformation of these cells into non-nucleated particles or scales, takes place irregularly and at no certain distance from the surface, as in the healthy epidermis. After a time the capillaries become enlarged, but this seems to be only the effect of the excessive and abnormal development of the cells which are dependent upon their contents for their supply of nutritive material.

As only one instance of the beneficial effects of this substance would be totally insufficient to establish its claim to be a specific, I asked Mr. Ord, the house-surgeon of St. Thomas's Hospital, if he would try its effects on some of the out-patients, as, if it did no good it could not possibly do harm, which he informs me he has done, with a satisfactory result. It is also with the view of further testing its efficacy that I have been induced to ask permission to insert the present communication in this widely-circulated periodical, hoping that, if any reader should think proper to try its effects upon the disease in question, he will give it a complete trial.

I may further add, that it seems to me no unreasonable inference, that if creosote is capable of destroying excessive or abnormal cell-growth in the dermic tissue, it may also do the same in analogous diseases of the mucous tissues, and therefore that it may possibly be applied with advantage in nasal and uterine polypi. In these cases I would not recommend it unless it could be kept in contact with the diseased part for a considerable length of time. I have even recommended a professional friend of mine, who has at this time a case of epithelial cancer under his care, to make a trial of it in this disease.

As this class of diseases, besides being distinguished by abnormal cell-growth of greater or less activity, is also attended with a disordered condition of the system, the local application of creosote would not of itself be likely to be of much service. In such cases it would require to be taken also internally, and probably to be persisted in for many months, according to the effect it might have upon the local disease or upon the general health. I may observe that I am by no means sancuine as to the beneficial effects of the remedy I am proposing in those diseases which are known by the term malignant. My expectations upon this point are far from rising to an extravagant elevation. I merely think from what I have seen of its action that it is just worthy of a fair trial, especially as these diseases are at present incurable, and as the remedy which I propose for their cure or relief, very different to many others employed for the same purpose, is incapable of doing any harm, should it fail to do any good. It is impossible for me to say how far creosote may have been used in the diseases for which I have proposed to employ it. I have not read of any case of the kind where it has been employed, and no one of the medical men that I have interrogated upon the subject knows of any instance of its employment in the same complaint, and with the same physiological view, as that I have advanced. About a month since I went to a medical man's house to ask him if he could furnish me with any cases of warts, &c., upon which he could employ creosote. A person not in the profession was present, who, hearing the conversation, said he had taken a little boy to a druggist's some time before to have something applied to a wart, which he believed was creosote, and which cured it. This is all that I have heard of the employment of this substance in any of the above named diseases.—*London Lancet.*

